# The monoamine stabilizer OSU6162 has anxiolytic-like properties and reduces voluntary alcohol intake in a genetic rat model of depression

**DOI:** 10.1038/s41598-021-91215-1

**Published:** 2021-06-04

**Authors:** Philippe A. Melas, Malin Wirf, Helder André, Nitya Jayaram-Lindström, Aleksander A. Mathé, Pia Steensland

**Affiliations:** 1 Center for Psychiatry Research, Department of Clinical Neuroscience, Karolinska Institutet & Stockholm Health Care Services, 11364 Stockholm, Sweden; 2 Center for Molecular Medicine, L8:00, Karolinska University Hospital, 17176 Stockholm, Sweden; 3 Department of Clinical Neuroscience, Division of Eye and Vision, St. Erik Eye Hospital, Karolinska Institutet, 17164 Stockholm, Sweden

**Keywords:** Preclinical research, Addiction, Anxiety

## Abstract

Alcohol use disorders (AUD) often co-occur with anxiety and depressive disorders, and anxiety often drives relapse during alcohol abstinence. Optimal AUD pharmacotherapies may thus need to target both excessive alcohol intake and elevated anxiety. (−)-OSU6162 (OSU) is a monoamine stabilizer that attenuates alcohol-mediated behaviors in both preclinical and clinical settings. However, OSU’s effect on anxiety-like behavior following long-term drinking remains unknown. To this end, we utilized a genetic rat model that exhibits increased anxiety- and depression-like behaviors (Flinders Sensitive Line; FSL) and their controls (Flinders Resistant Line; FRL). Using the novelty suppressed feeding (NSF) test, we evaluated anxiety-like behaviors (1) at baseline, (2) following long-term voluntary drinking and after 24 h of alcohol deprivation, and (3) following OSU administration in the same animals. At baseline, FSL animals displayed significantly elevated anxiety-like characteristics compared to FRL. Compared to alcohol-naïve animals, long-term drinking significantly reduced anxiety-like behaviors in FSL, without any significant effects in FRL animals. Compared to vehicle, OSU administration significantly reduced anxiety-like behaviors in alcohol-naïve FSL and long-term drinking FRL animals. While there was no significant difference in alcohol intake between FSL and FRL, OSU attenuated alcohol intake in both strains. Conclusively, in addition to the compound’s previously identified ability to suppress alcohol-mediated behaviors, OSU may also possess anxiolytic properties, warranting further clinical evaluation in both AUD and anxiety disorder settings.

## Introduction

A complex relationship exists between anxiety, stress and alcohol drinking, with alcohol having anxiolytic and stress-relieving effects but also acting as a stressor^1^. A high degree of comorbidity between depression, anxiety and alcohol use disorders (AUD) is also clinically well recognized^2^, and with depressive and anxiety disorders predicting the first incidence of AUD^3^. The relationship between the aversive emotional states leading to anxiety symptoms and the symptoms of AUD, includes the possibility of being one of mutually reinforcing each other or that of a dose–response relationship (i.e., between the severity of the anxiety symptoms and the level of alcohol consumption)^4,5^. It has thus been suggested that the development of optimal and more innovative treatments may need to adopt a transdiagnostic approach by examining and addressing the shared (neurobiological and behavioral) features of anxiety disorders and AUD^6^. However, although there are a number of drug classes approved for treating anxiety disorders^7^, there are only three FDA-approved drugs for AUD (acamprosate, disulfiram, naltrexone), including a fourth (nalmefene) in Europe, all of which have small effect sizes^8^. Importantly, none of these medications address the comorbidity between anxiety and alcohol use problems, which may involve the brain’s monoaminergic system^9^.

(−)-OSU6162 (OSU) is a compound that acts as a stabilizer of dopaminergic and serotonergic brain signaling, presumably through its action as a neutral antagonist and/or a weak partial agonist at dopamine D2 and serotonin 5-HT2A receptors^10–13^. OSU’s normalizing effect on psychomotor activity and striatal dopaminergic function^14,15^ provided the basis for its evaluation in treating disorders with an underlying dopaminergic dysregulation, including AUD. Specifically, OSU attenuates several alcohol-mediated behaviors in preclinical models, including voluntary alcohol consumption, cue-induced reinstatement of alcohol seeking and the alcohol deprivation effect^16,17^. In a combined clinical and laboratory study, OSU attenuated priming-induced craving and subjective liking of alcohol in patients with AUD^18^. In addition, it was found that OSU had no short-term negative effects on any of the cognitive domains assessed in patients with AUD, while actually improving certain higher order cognitive functions^19^. Collectively, these findings suggested that OSU may have beneficial treatment effects on both craving and cognition in AUDs.

However, no studies to date have examined OSU’s effect both on anxiety and alcohol intake following a history of long-term drinking. To this end, we evaluated the effects of OSU on anxiety-like levels and voluntary alcohol intake in a preclinical genetic model: the Flinders Sensitive Line (FSL) and their controls, the Flinders Resistant Line (FRL). The FSL line has traditionally been used as a genetic rat model of depression and also exhibits anxiogenic-like behaviors^20–24^. In view of the above, we used the FSL/FRL model and, by evaluating anxiety-like behaviors using the novelty suppressed feeding (NSF) test, we formulated the following three questions, previously not explored: (1) Are there differences in anxiety-like behaviors and/or levels of voluntary alcohol intake in FSL compared to FRL animals? (2) Does long-term alcohol drinking affect anxiety-like behaviors in the FSL/FRL model? (3) Does the monoamine stabilizer OSU affect anxiety-like behaviors and voluntary alcohol intake in the FSL/FRL model? Data from the present study provide the first evidence, to our knowledge, of OSU’s anxiolytic-like properties, including OSU’s ability to reduce voluntary alcohol intake, in a genetic rat model of depression.

## Materials and methods

### Animals

Male FSL (n = 20) and control-FRL (n = 20) rats were obtained from the colony maintained by AAM at the Karolinska Institutet. Animals were housed individually in Macrolon cages covered with filter tops (Tecniplast, Italy) under a reversed light/dark 12-h cycle (lights out at 9 a.m.). Food and water were available ad libitum, except prior to the novelty suppressed feeding (NSF) tests when food restrictions were applied, as described below. All behavioral tests and drinking measurements were carried out by an experimenter blind to the animal strains in a dark room illuminated by red lights. The Ethical Committee on Animal Research in Stockholm, Sweden approved all experimental procedures (Dnr N163/14). Experiments were carried out in accordance with all relevant guidelines and regulations, and in compliance with the ARRIVE guidelines.

### Locomotor activity tests

**Figure 1:**
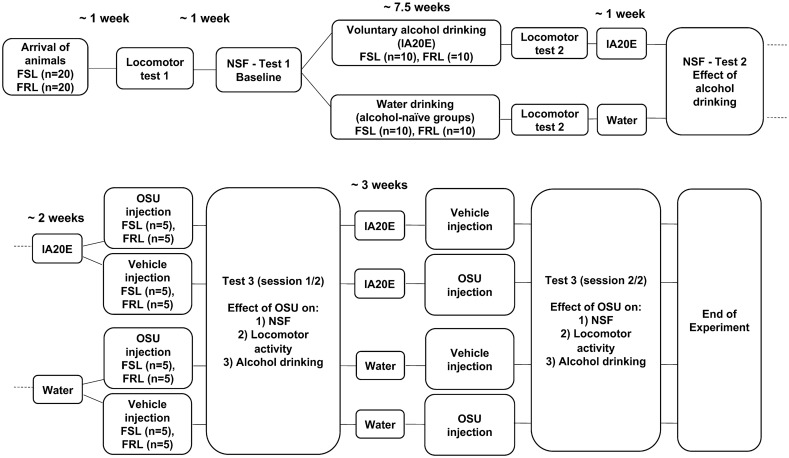
Schematic experimental timeline. A total of n = 20 control-FRL (the Flinders Resistant Line) and n = 20 FSL (the Flinders Sensitive Line—a genetic rat model of depression) animals were used. Following locomotor test 1 and NSF test 1, FSL and FRL animals were divided into two groups, i.e., one group that had access to alcohol under the IA20E protocol (n = 10 animals per FSL and FRL groups) and one that had access to water only (n = 10 animals per FSL and FRL groups). The NSF test 3 was conducted during two test sessions, i.e., OSU session 1/2 and OSU session 2/2, separated by approx. 3 weeks of access to IA20E or water. For NSF test 3, all animals were given both treatments [i.e., OSU and vehicle (saline)] and were randomized to receiving either OSU or vehicle at the first test session. Single OSU or vehicle injections were given 60 min before the start of NSF test 3. To evaluate the effect of OSU on voluntary alcohol intake, rats were given access to alcohol and water immediately after locomotor tests 3.

Locomotor activity in the FSL and FRL rats was measured using the open field test (a wooden square arena of 60 × 60 × 35 cm) at three time points: (1) Baseline locomotor activity following one week of acclimation and handling. During the test, rats were placed in the center of the arena and total distance travelled was recorded with the EthoVision XT10 software (Noldus, Noldus Information Technology, The Netherlands) for 30 min. (2) In order to examine whether long-term drinking affects locomotor activity the test was repeated one week before the NSF test 2 (see below). (3) In order to examine whether OSU administration affects locomotor activity, food and lights were removed immediately after the end of NSF test 3 (i.e., following both OSU sessions; see NSF test 3 below), and rats were kept in the arena for an additional 15 min to record locomotion. For a schematic timeline of the locomotor activity tests see also Fig. 1.

### Novelty suppressed feeding (NSF) tests

The NSF is a conflict-based test in which animals deprived of food are given the opportunity to approach and consume food in the center of an anxiogenic environment, i.e., a brightly lit open arena. The main measured variable is the latency to approach and/or eat the food—and administration of drugs with anxiolytic properties, e.g., benzodiazepines, decrease this latency in rodents^25^. Therefore, animals with significantly longer latencies are described as more anxious. Three NSF tests (see below) were used to assess anxiety levels in FSL and FRL animals that were food restricted for six hours. Food pellets were placed in the center of a brightly lit open field arena (same as the one used for locomotor tests) and the rat was placed in the corner of the arena. Measurements were taken of the latency (in s) to approach the food, the number of approaches and the latency to eat the food (in s) during a 20-min test period. Although all animals approached the food, not all animals chose to eat during the test period. Animals that chose not to eat were included in analyses of latency-to-approach and number of approaches but were excluded from analyses of latency-to-eat. For a schematic timeline of the NSF tests see also Fig. 1.

### NSF test 1—baseline measures of anxiety

The NSF test 1 was conducted to assess baseline anxiety levels in FSL and FRL animals, 1 week after the baseline locomotor activity assessments.

### NSF test 2—effect of long-term voluntary alcohol drinking on anxiety levels

Following the NSF test 1, half of the rats from each strain (FSL and FRL) were randomized to intermittent access to 20% ethanol (IA20E) or water (see also “Drinking model”, below). The alcohol-naïve rats were handled, weighed and single-housed in the same manner as the alcohol-drinking rats. The NSF test 2 was conducted following approximately 8 weeks of IA20E (or water), to assess alcohol-induced changes in anxiety-like levels. The alcohol groups were deprived of alcohol 24 h before the start of this testing. Water was always available ad libitum.

### NSF test 3—effect of OSU on anxiety levels

The NSF test 3 was conducted during two test sessions to evaluate the effect of OSU administration on anxiety-like levels. Specifically, following NSF test 2, animals were subjected to two weeks of IA20E (or water for the alcohol-naïve groups). All animals were given both treatments [i.e., OSU and vehicle (saline)] and were randomized to receiving either OSU or vehicle at the first test session. Between these two NSF 3 test-sessions, the rats had approximately three additional weeks of IA20E or water. Single OSU or vehicle injections were given 60 min before the start of the NSF test (see also “Drugs and chemicals”, below).

### Drinking model

We employed the intermittent access to 20% ethanol (IA20E) two-bottle-choice drinking model^16,26–28^. The IA20E model has been found, both by us and others, to induce behavioral and neurochemical changes that occur during the development of AUD^29–31^ and to be valuable in identifying potential novel AUD medications^16,32^. Successful clinical studies with OSU and varenicline in AUD patients provide support for the predictive validity of the IA20E model^18,33^. A detailed description of the IA20E protocol has been published in our previous work^16^. Briefly, rats had access to alcohol during three 24-h sessions per week (Monday, Wednesday and Friday), each beginning ~ 10 min following lights-off. Water was always available ad libitum. To examine differences in alcohol intake between FSL and FRL, we assessed alcohol intake levels (g/kg/24 h) at all 33 sessions of the IA20E drinking model leading up to the OSU experiment. To evaluate the effect of OSU on voluntary alcohol intake (g/kg/1 h and g/kg/24 h), rats were given access to alcohol and water immediately after the locomotor test 3, which followed the NSF test 3. The alcohol-naïve groups were housed and handled under identical conditions with the exception that they had continuous access to two bottles of water.

### Drugs and chemicals

Drinking solutions were prepared in tap water from 95% (v/v) ethanol (Solveco AB, Sweden). The monoamine stabilizer (−)-OSU6162 [(S)-(−)-3-(3-methanesulfonyl-phenyl)-1-propyl-piperidine] (PNU-96391; OSU) was dissolved in 0.9% saline and administered subcutaneously at a dose of 30 mg/kg body weight (injection volume: 5 ml/kg). The OSU dose was based on our previous rat studies, showing that the chosen dose of 30 mg/kg attenuates several alcohol-mediated behaviors without inducing any motor or memory impairments, and without any reinforcing properties^16,17,30^. Human safety studies have found that orally administered OSU is rapidly absorbed and well tolerated at doses ranging from 1 to 150 mg, maximum concentrations are achieved between 0.5 and 4 h, and the drug has a half-life of 2–6 h^34^. Besides having well-documented behavioral effects on alcohol-mediated behaviors, the single OSU dose of 30 mg/kg compared to vehicle (with a within-subject design) in the present study, was favored compared to a full dose–response study due to ethical considerations and the ambition to reduce the number of animals used in line with the principles of the 3Rs.

### Statistical analyses

For FSL versus FRL comparisons, normality of the data was examined using the Shapiro–Wilk test, and comparisons were performed using two-tailed (paired or unpaired) Student's t-tests and Mann–Whitney tests for normally and non-normally distributed data, respectively. The effects of alcohol intake and OSU administration on anxiety-like levels and locomotor activity in FSL and FRL animals, including differences in alcohol intake between groups, were assessed using two-way ANOVAs, two-way repeated measures ANOVAs or mixed models, followed by correction for multiple testing using Sidak's multiple comparisons test. The repeated measures/mixed ANOVAs were used to take into account the repeated testing in experiments with a within-subjects design. The effects of OSU administration on alcohol intake were analyzed using paired t-tests. Due to omission of taking the baseline values obtained in NSF 1 into account when randomizing the rats to the alcohol-exposure or alcohol-naïve groups, the results from the NSF2 experiment is presented as % change from baseline. Data are presented as mean values and graph error bars represent standard error of the mean (SEM). Outliers were identified using the ROUT test (Q = 1%) and were excluded from the statistical analyses. The number of animals used for each statistical analysis, including the number of identified outliers, is denoted in the corresponding figure legend. Statistical significance was set at P ≤ 0.05 and all analyses were performed using GraphPad Prism 8 (GraphPad Software, Inc., La Jolla, CA, USA).

## Results

### NSF test 1: increased anxiety-like behaviors in FSL animals at baseline

**Figure 2:**
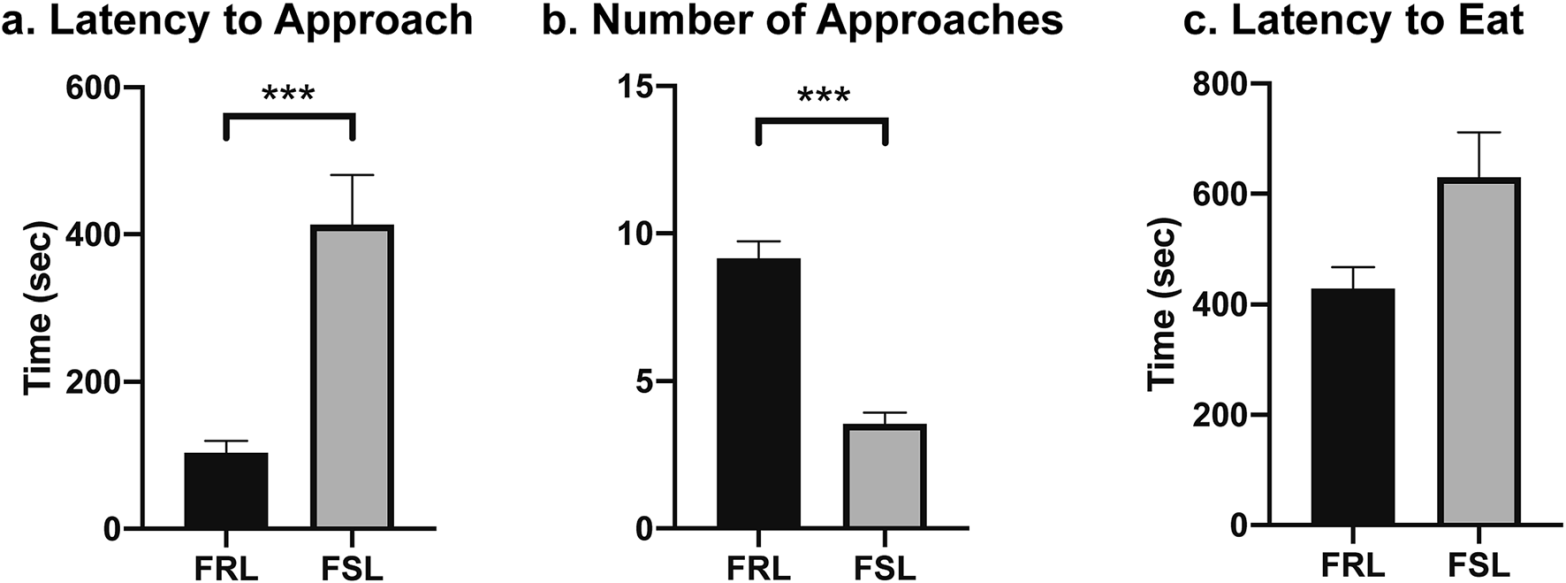
FSL animals display increased anxiety-like characteristics at baseline. The Novelty Suppressed Feeding test (NSF) was used to assess anxiety-like behavior at baseline (NSF test 1). Compared to the control FRL, the FSL rats displayed (**a**) significantly higher latency to approach the food (n = 19–20 animals/group, n = 1 outlier) and (**b**) significantly lower number of approaches (n = 20 animals/group). However, (**c**) there was no significant difference in the latency to eat the food between the two rat strains (n = 17–18 animals/group). Graph data are presented as mean ± SEM and were analyzed by Mann Whitney tests. ***P < 0.001.

First, we confirmed that FSL animals exhibit increased baseline anxiety-like levels compared to FRL animals. Specifically, in the NSF test (NSF test 1; see also Fig. 1 for a schematic timeline of the experiments) and compared to FRL, FSL animals showed significantly increased latency to approach the food (Fig. 2a; Mann Whitney test; Median: FRL = 78, FSL = 366; Mann–Whitney U = 35; P < 0.0001) and reduced number of approaches (Fig. 2b; Mann Whitney test; Median: FRL = 9, FSL = 3; Mann–Whitney U = 12.5; P < 0.0001). However, the difference in latency to eat the food was not statistically significant between groups (Fig. 2c; Mann Whitney test; Median: FRL = 432, FSL = 458; Mann–Whitney U = 114; P = 0.20). Moreover, there was no significant difference between FSL and FRL in levels of locomotor activity (locomotor test 1, unpaired t-test; t = 0.17; df = 37; P = 0.86; data not shown).

### No difference in alcohol intake between FSL and FRL animals

**Figure 3:**
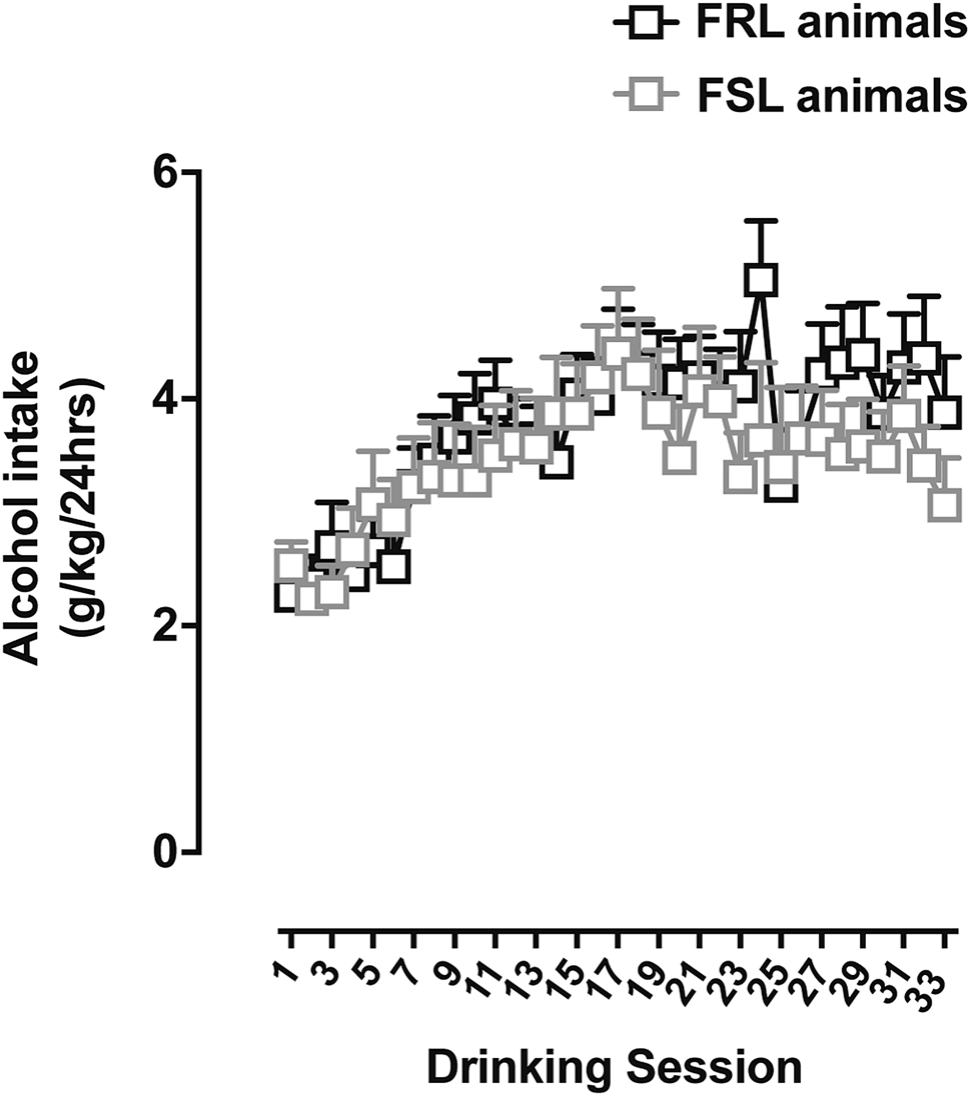
Alcohol intake levels in FSL and FRL animals. Animals had voluntary access to alcohol for a total of n = 33 IA20E alcohol sessions prior to the OSU experiment. Both FSL and control-FRL animals escalated significantly their alcohol intake as the result of time/drinking session. However, there was no significant difference in alcohol intake between FSL and controls at any given session (n = 10 animals per group; n = 1 outlier alcohol session). Graph data are presented as mean ± SEM and were analyzed by a mixed-effects model.

There was a significant escalation in voluntary alcohol intake in both FSL and FRL animals as a result of the long-term IA20E alcohol protocol, but with no significant differences in alcohol intake between the two rat strains (Fig. 3; Mixed-effects model: Time, F(32.00, 555.0) = 8.80, P < 0.0001; Animal strain: F(1, 18) = 0.28, P = 0.60; Interaction, F(32, 555) = 1.09, P = 0.33).

### NSF test 2: long-term drinking reduces anxiety-like behaviors in FSL animals

**Figure 4:**
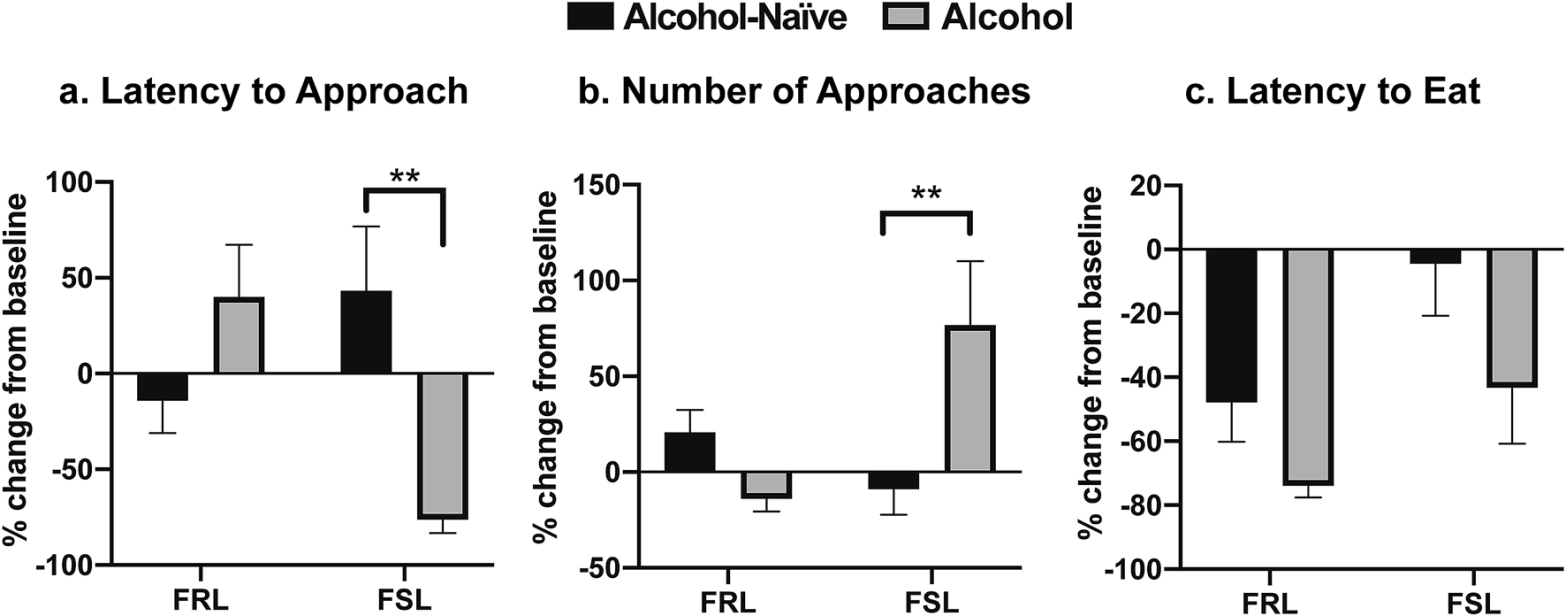
Long-term drinking decreases anxiety-like characteristics in FSL animals. The Novelty Suppressed Feeding (NSF) test was repeated following > 8 weeks of voluntary drinking according to the IA20E protocol to assess alcohol-associated changes in anxiety-like behaviors (NSF test 2). Analyses of the percentage change from the pre-alcohol baseline (i.e., NSF test 1), revealed anxiolytic-like effects of alcohol in the FSL rats as evidenced by (**a**) a significant reduction in the latency to approach the food (n = 9–10 animals/group, n = 2 outliers) and (**b**) a significant increase in the number of approaches (n = 9–10 animals/group, n = 1 outlier). However, (**c**) no significant alcohol-induced changes were found in the latency to eat (n = 6–9 animals/group, n = 1 outlier). (**a**–**c**) In the control FRL-group there were no significant alcohol-induced changes compared to pre-alcohol baseline in any of the analyzed behaviors. Graph data are presented as mean ± SEM and were analyzed by two-way ANOVA followed by Sidak’s multiple comparisons test. **P < 0.01.

Next, using the long-term IA20E voluntary drinking protocol, we first asked how alcohol use affects anxiety-like behaviors in FSL and FRL rats (NSF test 2; assessed 24 h after the end of the last ethanol session prior to the test). Long-term drinking had anxiolytic-like effects in FSL animals, as evidenced by a significant decrease in the latency to approach the food [Fig. 4a; two-way ANOVA: Interaction, F(1, 34) = 12.71, P = 0.001; Sidak’s multiple comparisons test, alcohol-naïve versus alcohol drinking, P = 0.002] and a significant increase in the number of approaches, compared to the pre-alcohol baseline [Fig. 4b; two-way ANOVA: Interaction, F(1, 35) = 9.501, P = 0.004; Sidak’s multiple comparisons test, alcohol-naïve versus alcohol, P = 0.008]. However, there was no significant alcohol-induced change in the latency to eat the food [Fig. 4c; two-way ANOVA: Interaction, F(1, 28) = 0.18, P = 0.67]. In FRL-control animals, voluntary long-term drinking did not produce any significant anxiolytic- or anxiogenic-like effects compared to the pre-alcohol baseline (Fig. 4a–c; Sidak’s multiple comparisons test, alcohol-naïve versus alcohol drinking, P > 0.2 for all FRL comparisons). Within the FSL or FRL groups, alcohol drinking did not significantly affect locomotor activity levels [locomotor test 2, two-way ANOVA: Interaction, F(1, 36) = 0.58, P = 0.44; data not shown].

### NSF test 3: the monoamine stabilizer OSU is anxiolytic in alcohol-naïve FSL and long-term alcohol-drinking FRL animals

Finally, in the NSF test 3, we evaluated the effect of the monoamine stabilizer OSU on anxiety-like behaviors both in long-term alcohol-drinking FSL/FRL rats (following 24 h of alcohol deprivation) and in alcohol-naïve animals.

**Figure 5:**
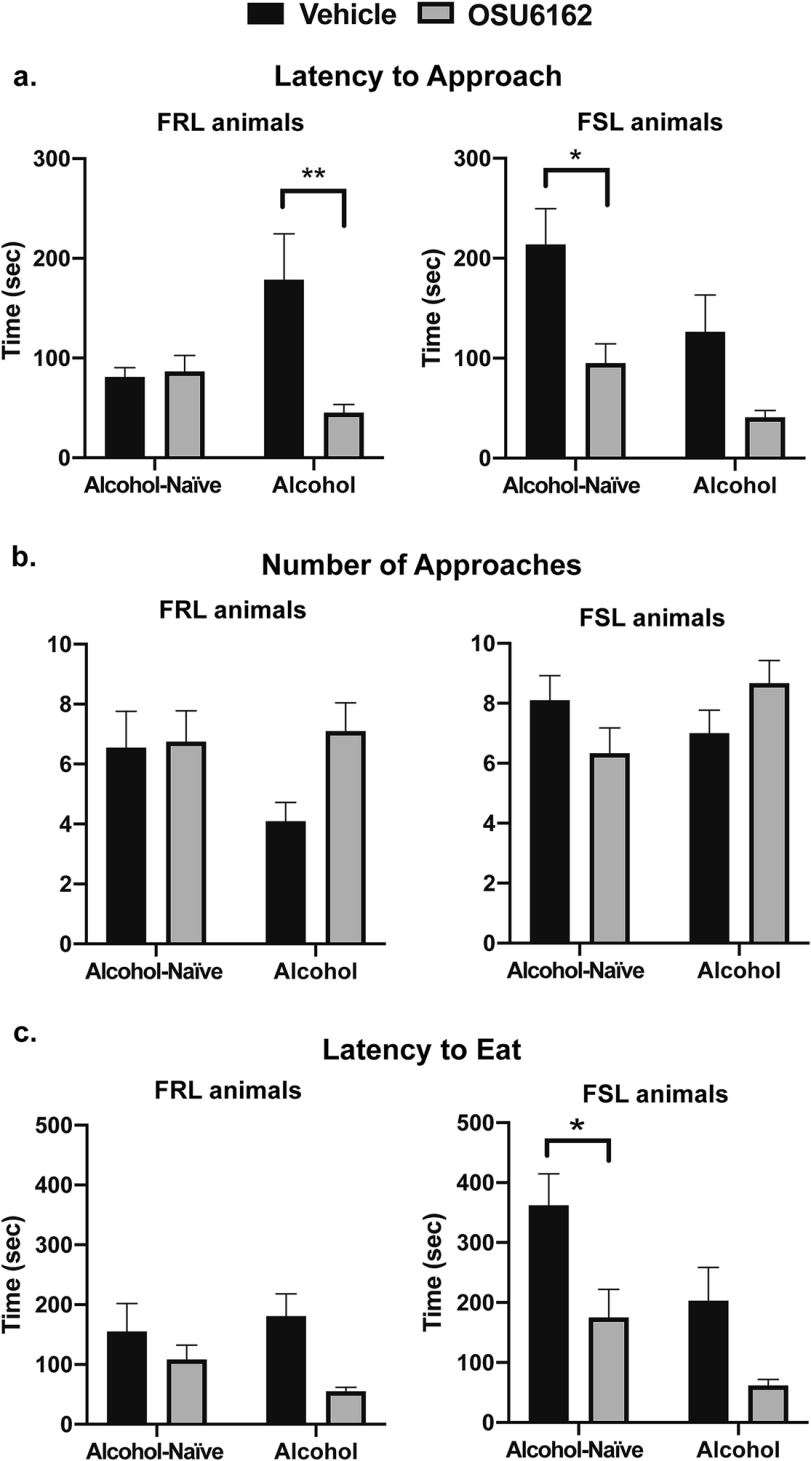
OSU6162 has anxiolytic-like properties. The Novelty Suppressed Feeding (NSF) test was used to evaluate the effects of the monoamine stabilizer OSU6162 (OSU) on anxiety-like behaviors in long-term alcohol drinking and alcohol-naïve rats from the FSL and their controls FRL. The NSF test 3 was repeated twice to allow all animals to receive both treatments: OSU (30 mg/kg) or vehicle (saline); see also schematic experimental timeline (Fig. 1). Treatments were administered subcutaneously 60 min before the NSF test and the rats were randomized to receiving either OSU or vehicle at the first test occasion. (**a**) In control FRL animals, OSU had a significant main effect on latency to approach the food and interacted with alcohol exposure, and post-hoc assessments showed a significant difference in latency between OSU and vehicle in the alcohol group (n = 8–9 animal pairs/group; n = 1 outlier). In FSL animals, both alcohol exposure and OSU administration had significant main effects on the latency to approach the food, and post-hoc assessments showed a significant difference in latency between OSU and vehicle in the alcohol-naïve group, and a close-to-significant difference in the alcohol group (n = 8–9 animal pairs/group; n = 1 outlier). (**b**) OSU had no significant effect on the number of approaches in controls (n = 8–10 animal pairs/group) or in FSL animals (n = 9 animal pairs/group). (**c**) In controls, OSU had a significant main effect on the latency to eat the food, with post-hoc assessments revealing a close-to-significant difference between OSU and vehicle in the alcohol group (n = 3–6 animal pairs/group; n = 1 outlier). In addition, in FSL animals, both alcohol exposure and OSU administration had significant main effects on latency-to-eat, with post-hoc assessments showing a significant difference in latency between OSU and vehicle in the alcohol-naive group, and a trend in the alcohol group (n = 7–8 animal pairs/group; n = 1 outlier). Graph data are presented as mean ± SEM and were analyzed by mixed-effects models followed by Sidak’s multiple comparisons test. *P < 0.05, **P < 0.01.

In FSL animals, both alcohol exposure and OSU administration had significant main effects on latency-to-approach, with post-hoc assessments showing a significant difference between OSU and vehicle in the alcohol-naïve group, and a close-to-significant difference in the alcohol group [Fig. 5a; Mixed-effects model: Alcohol treatment, F(1, 18) = 5.13, P = 0.03; OSU treatment, F(1, 15) = 13.61, P = 0.002, Interaction, F(1, 15) = 0.40, P = 0.53; Sidak's multiple comparisons test, Vehicle—OSU6162: Alcohol-naïve group, P = 0.01; Alcohol group, P = 0.08]. However, OSU had no significant effect on the number of approaches although there was a weak interaction between the OSU and alcohol groups [Fig. 5b; Mixed-effects model: Alcohol treatment, F(1, 34) = 0.58, P = 0.44; OSU treatment, F(1, 34) = 0.003, P = 0.95, Interaction, F(1, 34) = 4.54, P = 0.04; Sidak's multiple comparisons test, Vehicle—OSU6162: Alcohol-naïve group, P = 0.24; Alcohol group, P = 0.28]. Moreover, both alcohol exposure and OSU administration had significant main effects on latency-to-eat, with post-hoc assessments showing a significant difference in latency between OSU and vehicle in the alcohol-naive group, and a trend in the alcohol group [Fig. 5c; Mixed-effects model: Alcohol treatment, F(1, 30) = 7.76, P = 0.009; OSU treatment, F(1, 30) = 11.23, P = 0.002, Interaction, F(1, 30) = 0.21, P = 0.64; Sidak's multiple comparisons test, Vehicle—OSU6162: Alcohol-naïve group, P = 0.02; Alcohol group, P = 0.10].

In the NSF testing of FRL rats, OSU had a significant main effect on latency-to-approach and interacted with alcohol exposure, with post-hoc assessments showing a significant difference between OSU and vehicle in the alcohol group [Fig. 5a; Mixed-effects model: Alcohol treatment, F(1, 17) = 0.97, P = 0.33; OSU treatment, F(1, 15) = 5.89, P = 0.02, Interaction, F(1, 15) = 6.88, P = 0.01; Sidak's multiple comparisons test, Vehicle—OSU6162: Alcohol group, P = 0.004]. Although, OSU had no significant effect on the number of approaches [Fig. 5b; Mixed-effects model: Alcohol treatment, F(1, 17) = 0.99, P = 0.33; OSU treatment, F(1, 16) = 4.45, P = 0.05, Interaction, F(1, 16) = 2.985, P = 0.10], the compound had a significant main effect on latency-to-eat, and with post-hoc assessments revealing a close-to-significant difference between OSU and vehicle in the alcohol group [Fig. 5c; Mixed-effects model: Alcohol treatment, F(1, 16) = 0.05, P = 0.81; OSU treatment, F(1, 7) = 9.39, P = 0.01, Interaction, F(1, 7) = 0.6193, P = 0.45; Sidak's multiple comparisons test, Vehicle—OSU6162: Alcohol-naïve group, P = 0.21; Alcohol group, P = 0.08].

OSU had no significant effect on levels of locomotor activity (locomotor test 3) in FSL animals [two-way repeated measures ANOVA: Alcohol treatment, F(1, 32) = 0.27, P = 0.60; OSU treatment, F(1, 32) = 2.95, P = 0.09, Interaction, F(1, 32) = 0.04, P = 0.83; data not shown] or in FRL-controls [two-way repeated measures ANOVA: Alcohol treatment, F(1, 17) = 0.64, P = 0.43; OSU treatment, F(1, 17) = 1.16, P = 0.29, Interaction, F(1, 17) = 0.17, P = 0.68; data not shown].

### OSU reduces voluntary alcohol intake

**Figure 6:**
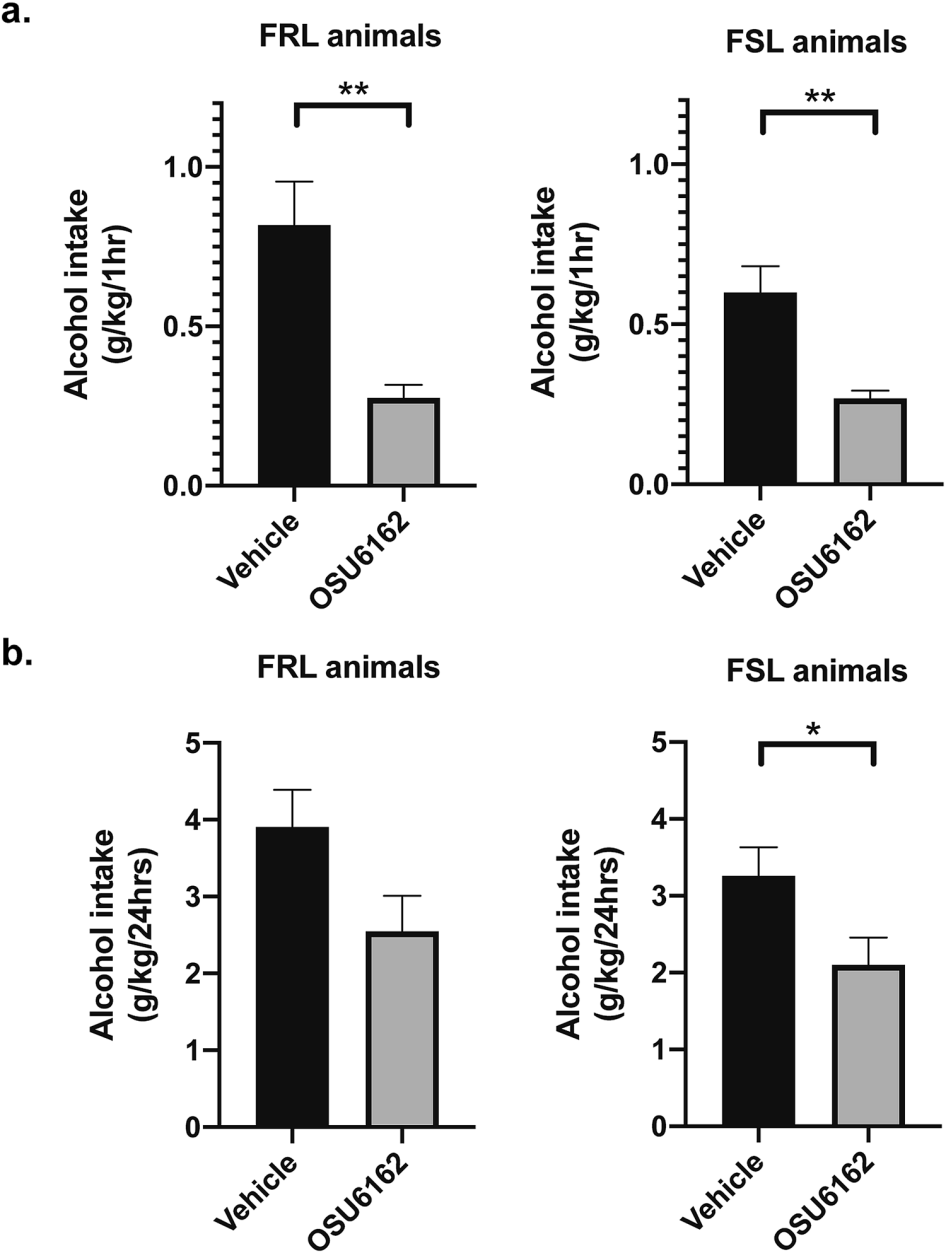
The effects of OSU administration on alcohol intake in FSL and FRL animals. (**a**) Single OSU6162 (OSU; 30 mg/kg) administration, 60 min before the start of NSF test 3 (see also Fig. 1), significantly reduced alcohol intake both in FSL and FRL-control animals at 1 h into the alcohol session (n = 9 animal pairs/group). (**b**) The effect of OSU administration on reducing alcohol intake remained significant in FSL animals, but not in FRL-controls, at 24 h into the alcohol session (n = 9 animal pairs/group). Graph data are presented as mean ± SEM and were analyzed by paired t-tests. *P ≤ 0.05, **P < 0.01.

When we examined the effect of a single OSU injection on voluntary alcohol intake, we found that compared to vehicle, OSU significantly decreased alcohol intake in both FSL and FRL animals at 1 h into the alcohol session (Fig. 6a; FSL: paired t-test, t = 3.471, df = 8, P = 0.008; FRL: paired t-test, t = 3.582, df = 8, P = 0.007). In addition, the effect of OSU on reducing alcohol intake remained significant at 24 h into the alcohol session in the FSL, but not the FRL, rats (Fig. 6b; FSL: paired t-test, t = 2.207, df = 8, P = 0.05; FRL: paired t-test, t = 1.999, df = 8, P = 0.08).

## Discussion

Alcohol use disorder is prevalent among individuals suffering from anxiety and depression^35^, and although the underlying mechanisms resulting in these comorbidities have not been fully clarified, there is strong evidence supporting an involvement of the brain’s monoaminergic system^9^. This relationship between AUD, anxiety/depression and monoamines, is supported by the present study’s most salient findings which include the following: (a) The depressed FSL line displays increased baseline anxiety-like behaviors compared to FRL-controls, (b) Long-term voluntary drinking reduces anxiety-like behaviors in FSL rats undergoing alcohol deprivation, (c) the monoamine stabilizer OSU significantly reduces anxiety-like behaviors in alcohol-naïve FSL and long-term alcohol-drinking FRL rats, and (d) OSU reduces voluntary alcohol intake in both FSL and FRL rats.

Specifically, we found that FSL animals display an anxious-like phenotype at baseline compared to FRL rats, which was evidenced by increased latency to approach the food and reduced number of approaches in the first NSF test. This is in line with previous studies demonstrating an anxiety-like phenotype in FSL rats using other anxiety-related tests, such as the social interaction test, the light/dark box test and the active avoidance task^21–23^. We found that long-term voluntary alcohol drinking significantly decreased the anxiety-like behaviors in FSL animals, as assessed 24 h after the last ethanol session prior to the test. Although there was no significant effect of long-term drinking on anxiety-like behaviors in FRL rats, visual inspection of Fig. 4a,b showed a reversed behavioral pattern between the two rat strains, suggesting that alcohol may have opposite effects on anxiety-like behavior in FSL and FRL rats. Contrary to our expectations, however, we did not observe enhanced alcohol drinking in the FSL animals, which may reflect the presence of FSL/FRL strain differences in alcohol pharmacodynamics. Indeed, compared to the FRL line, FSL animals have been found to exhibit a greater degree of alcohol-induced hypothermia, including slightly higher blood ethanol concentrations following an IP injection with 1.5 g/kg ethanol^36^. The FSL-specific finding of decreased anxiety-like levels found in the present study following long-term drinking, together with a putatively higher blood ethanol concentrations in FSL compared to FRL^36^, may thus potentially provide support to the self-medication hypothesis where alcohol use is driven by an urge to alleviate anxiety^37^.

In the present study, we also found that OSU reduced voluntary alcohol intake in both FSL and FRL rats. These results are in line with previous rodent findings showing that OSU attenuates a number of alcohol-mediated behaviors, including alcohol intake, alcohol self-administration under a progressive ratio reinforcement schedule, and cue-induced reinstatement of alcohol seeking in Wistar rats^16^. Previous preclinical studies have also found that OSU prevents the alcohol deprivation effect, i.e., relapse-like drinking following abstinence in long-term drinking rats^17^. A subsequent human laboratory study demonstrated that treatment with OSU blunted the subjective liking of alcohol and reduced priming-induced alcohol craving in alcohol dependent individuals^18^. Given the high comorbidity between AUD and anxiety, and the putative involvement of the brain’s monoaminergic system in both disorders^9^, we also examined the effect of OSU on anxiety-like behaviors in alcohol-naïve as well as in long-term drinking FSL and FRL rats. The results showed that, compared to vehicle, OSU had significant anxiolytic-like effects in the alcohol-naïve FSL rats and in the long-term alcohol-drinking FRL rats. The lack of significant anxiolytic-like effects of OSU in the alcohol-drinking FSL, which is depicted in the right panels of Fig. 5a,c, appears to indicate that a lower threshold of anxiety-like behavior was achieved in the FSL rats due to the long-term voluntary drinking. However, without a full OSU dose–response analysis (which was omitted in the present study due to ethical considerations), this hypothesis is difficult to evaluate. Still, the present data provide novel preclinical evidence for OSU’s concurrent suppressing effects on alcohol intake and anxiety-like behaviors, and support OSU’s future evaluation as a potential medication in patients with anxiety disorders with or without co-occurring AUD. Given the shared genetic factors of the two disorders, future clinical studies should also consider the systematic collection of family history data on AUD to assess the prospective effects of OSU on alcohol use in individuals with an anxiety disorder and a family history of AUD, but without a current AUD diagnosis.

Future studies are also necessary to elucidate the neurobiological mechanisms that underlie OSU’s therapeutic-like effects on anxiety-like behaviors. We hypothesize that dopaminergic stabilization is the primary mechanism of OSU’s action, based on its ability to act at dopamine D2 receptors and to counteract both hypo- and hyperdopaminergic states^10,11,13^. The dopaminergic system plays a critical role in anxiety-like behaviors^38–40^ and in the development and maintenance of AUD^31,41,42^. OSU has been found to target extrasynaptic dopamine D2/D3 receptors in the striatum^12^ and a microdialysis study showed that it has the ability to counteract dopamine deficits in the nucleus accumbens of long‐term drinking rats, possibly by acting as an antagonist at presynaptic D2 autoreceptors^16,30^. Thus, since OSU had significant anxiolytic effects in alcohol-naïve FSL animals, this may indicate that the FSL model harbors genetically-driven dopaminergic deficits that contribute to its anxious-like phenotype at baseline. Indeed, relative to FRL rats, the FSL model has been found to display various neurochemical abnormalities, including in the dopaminergic, glutamatergic and neuropeptidergic systems^21,43–47^. With regard to the dopaminergic system, FSL animals are known to display sensitivity toward dopamine agonists, yet without any apparent brain changes in dopamine receptor concentrations^48^. However, serotonergic abnormalities and significantly decreased dopamine release from nucleus accumbens following serotonin application, point to a dysregulated serotonergic-dopaminergic interaction in the FSL model^49–51^. It is also worth noting that, in addition to the modulation of the dopaminergic system, OSU has been found to bind to the sigma-1 receptor^52^, which may serve as another candidate for OSU’s anxiolytic and alcohol-suppressing properties, given the role of sigma receptors in anxiety and alcohol dependence^53,54^.

Finally, as part of our study’s limitations, it should be noted that (1) each rat was subjected to the NSF experiment on repeated occasions, possibly reducing the novelty parameter of the experiment at each session. However, due to ethical considerations, our study was designed to minimize the number of experimental animals used, by allowing for within-subject comparisons. Furthermore, several weeks were allowed between each NSF session to minimize the potential risk of lack of novelty between the different test sessions and there was no sign of habituation, indicated by no significant differences in locomotor activity levels between pre- and post-drinking experiments (data not shown). Moreover, (2) although our findings provide the first evidence for OSU’s anxiolytic-like properties using the NSF test, future studies are warranted to investigate OSU’s efficacy in additional anxiety models, e.g., in ethological versus conditioned operant conflict tests, including protocols used to induce stress responses^55^. In addition, although FSL animals display anxiogenic behaviors in some tests, such as the NSF (presented here), the social interaction test, the active avoidance task, and the light/dark box test, they have not been found to differ from FRL animals in the elevated plus maze^20–24^. This may indicate the presence of genetic variations in the FSL/FRL model that confer specificity to certain anxiety-like behaviors. Finally, (3) the small animal subgroups, present in some of our comparisons, made statistical interactions difficult to detect and/or interpret. In addition, we evaluated male animals only, and future studies are needed to examine the anxiolytic-like effects of OSU in female cohorts and by taking into account the sex-dependent differences found in human populations. Specifically, both anxiety and depressive disorders are overrepresented in women^56^ and the relative risk of individuals with anxiety disorders presenting with increased risk for alcohol abuse is higher in women than in men^57^.

## Conclusion

The monoamine stabilizer OSU6162 is a compound with favorable clinical tolerability and with the ability to increase or decrease dopaminergic signaling depending on the endogenous tone^14,15,58^. The compound has shown promising preclinical and clinical results in AUD settings by attenuating alcohol-mediated behaviors^18,19^. More recently, a preclinical study also reported OSU-induced reductions in opioid craving and relapse^59^. The present findings replicate the efficacy of OSU in reducing alcohol intake and provide the first preclinical data, to the best of our knowledge, demonstrating OSU’s anxiolytic-like properties. Given the compound’s safety profile, the present study suggests that OSU’s evaluation in clinical settings of anxiety disorders, with or without comorbid AUD, is warranted.
